# Association between Oral Contraceptive Use and the High-Sensitivity C-Reactive Protein Level in Premenopausal Korean Women

**DOI:** 10.3390/healthcare10020361

**Published:** 2022-02-12

**Authors:** Hyejin Park

**Affiliations:** Department of International Healthcare Administration, Daegu Catholic University, Gyeongsan 38430, Korea; hjpark@cu.ac.kr; Tel.: +82-53-850-2593

**Keywords:** oral contraceptive, high-sensitivity C-reactive protein, inflammation, premenopausal women, KNHANES

## Abstract

Although oral contraceptives (OCs) are widely used, few national epidemiological studies have evaluated the association between OC use and serum high-sensitivity C-reactive protein (hs-CRP) levels in Korean women. This population-based cross-sectional study was conducted with data from the 2015–2018 National Health and Nutrition Examination Survey. In the sample of 5332 premenopausal women aged ≥19 years, hs-CRP concentrations were 1.087 mg/L among OC users and 0.953 mg/L among OC non-users. After adjustment for confounders, OC users had an increased likelihood of having risky (>1.0 mg/L) hs-CRP levels (adjusted odds ratio (aOR) = 1.58; 95% confidence interval (CI), 1.25–1.98) compared with OC non-users. In addition, the aOR for high-risk (>3.0 mg/L) hs-CRP levels in OC users compared with non-users was 1.51 (95% CI, 1.06–2.16). These findings demonstrate that OC use alters the concentration of hs-CRP, a biomarker of chronic low-grade inflammation, and suggest that long-term OC use is a risk factor in the pathogenesis of inflammatory diseases, including cardiovascular diseases.

## 1. Introduction

With the recent expansion of access to contraception and a decrease in unmet needs for family planning, the worldwide prevalence of contraceptive use among women of childbearing age has increased to about 60% [1]. Oral contraceptives (OCs) are the most widely used contraception method worldwide [2]; more than 150 million women around the world currently use OCs as an effective, convenient family planning method [3,4]. However, many studies have documented associations between OC use and various side effects, including cardiovascular diseases (CVDs) such as arterial and venous thromboembolism, myocardial infarction, and ischemic stroke [5,6,7].

Although the mechanisms underlying the association between OC use and the incidence of CVDs have not been elucidated in detail, there is some evidence that OC use leads to chronic low-grade inflammation, as indicated by increases in high-sensitivity C-reactive protein (hs-CRP) concentrations [8,9,10]. As chronic low-grade inflammation is an important mechanism in the pathophysiology of CVD [11], an increase in the hs-CRP concentration implies a potential risk of CVD development [12,13]. The American Heart Association and US Centers for Disease Control and Prevention defined hs-CRP levels > 3.0 mg/L as reflecting a high CVD risk, levels of 1.0–3.0 mg/L as reflecting an average risk, and levels < 1.0 mg/L as reflecting a low CVD risk [14,15].

CVDs are major causes of death and disability worldwide [16]. In Korea, their prevalence has been decreasing in recent years, but they remain the second leading cause of death [17]. Thus, the identification of factors affecting the prevalence of CVD in the general population is essential for a reduction in premature death and disability due to CVD. Few studies have been conducted to examine the association between OC use and the hs-CRP level as an indicator of CVD risk in Korean women of childbearing age. Thus, this study was performed to assess this association in premenopausal Korean women using data from the Korea National Health and Nutrition Examination Survey (KNHANES).

## 2. Methods

### 2.1. Study Participants

This study was performed with raw data from the 2015–2018 KNHANES, provided by the Korea Disease Control and Prevention Agency. The KNHANES is conducted using a stratified multistage cluster sampling design, with proportional allocation based on the National Census Registry, to obtain a national representative sample of the Korean population. For this study, data from a sample of 5332 premenopausal women aged ≥19 years who were not pregnant were examined. The Korean Ministry of Health and Welfare approved the study protocol, and the study was performed in line with the tenets of the Declaration of Helsinki. All participants provided informed consent.

### 2.2. Data Collection

For the KNHANES, a well-structured questionnaire was used to collect data on respondents’ demographic and socioeconomic characteristics, including age, sex, education level, income, smoking habit, and alcohol consumption. Education levels were classified as below high school, high school, and above high school. Data on alcohol consumption were obtained with questions about respondents’ drinking behavior (average frequency of alcoholic beverage consumption) in the month before the interview. Respondents who reported current smoking and drinking at least once a month were classified as smokers and drinkers, respectively. After measuring the participants’ height and weight, the body mass index (BMI) was calculated by dividing the weight (in kilograms) by the square of the height (in meters). Subjects were then categorized as underweight (BMI < 18.5 kg/m^2^), normal weight (18.5 kg/m^2^ ≤ BMI < 23.0 kg/m^2^), overweight (23.0 kg/m^2^ ≤ BMI < 25.0 kg/m^2^), and obese (BMI ≥ 25.0 kg/m^2^) based on the World Health Organization’s definitions for Asian populations. Serum hs-CRP values were obtained using a COBAS analyzer (Roche Diagnostics) and an immunoturbidimetric assay (Roche Cardiac C-Reactive Protein High Sensitive reagent kit). The analytic measurement range for hs-CRP concentrations was 0.1–20 mg/L. Risky and high-risk hs-CRP levels were defined as those >1.0 mg/L and >3.0 mg/L, respectively.

### 2.3. Statistical Analysis

To describe the sample population, frequencies (including percentages) were calculated for categorical variables and means and standard deviations or standard errors were calculated for continuous variables. The *t* test was used to compare continuous variables between OC users and non-users. The significance of differences for categorical data was determined using the Mantel–Haenszel chi-squared test. Logistic regression models were used to estimate odds ratios (ORs) with 95% confidence intervals (CIs) for risky and high-risk hs-CRP statuses [vs. low risk (hs-CRP <1.0 mg/L)] in OC users compared with the reference group (OC non-users). Multivariate logistic regression was also used to estimate the association between OC use and risky or high-risk hs-CRP status, after adjusting for potential confounders such as age, BMI, education level, income, smoking habit, and alcohol consumption. The statistical analyses were performed with accounting for the survey design, and appropriate procedures in SAS (“surveylogistic”) were used with weighted data. All statistical analyses were conducted using SAS (ver. 9.4; SAS Institute, Cary, NC, USA).

## 3. Results

The study sample comprised 5332 women aged ≥19 years; their demographic characteristics are shown in Table 1. The mean age of the study population was 37.1 years and the BMI was 22.8 kg/m^2^.

Of the 5332 participants, 13.1% used OCs. The participants’ basic characteristics and the study outcomes are presented according to OC use in Table 2. OC use decreased with increasing education level (*p* < 0.001). Cigarette smoking was significantly associated with OC use (*p* < 0.001). OC use was not associated significantly with participants’ age, BMI, income, or alcohol consumption. The hs-CRP level was significantly higher among OC users than among OC non-users (1.098 vs. 0.953 mg/L, *p* < 0.05).

Risky (>1.0 mg/L) hs-CRP levels were found in 26.9% of OC users and 20.2% of OC non-users (*p* < 0.001). hs-CRP levels posing a high risk of CVD (>3.0 mg/L) were found in 9.0% of OC users and 6.4% of OC non-users (*p* = 0.011; Table 3).

Table 4 shows the ORs for the associations of risky and high-risk hs-CRP levels with OC use. The crude and adjusted odds ratios (aORs) for risky hs-CRP levels correlated positively with OC use (*p* < 0.001). Compared with OC non-use, OC use carried an aOR of 1.58 (95% CI, 1.25–1.98). Crude ORs and aORs for OC use were also significantly increased for participants with high-risk hs-CRP levels (*p* < 0.05). The crude OR for risky hs-CRP levels in the OC use group relative to the reference group (hs-CRP level < 1.0 mg/L) was 1.65 (95% CI, 1.18–2.29), and the aOR for high-risk hs-CRP levels was 1.51 (95% CI, 1.06–2.16).

## 4. Discussion

The use of OCs containing estrogen and progestin or progestin alone has long been an effective birth control method. Third- and fourth-generation OCs have been developed with improved safety and efficacy relative to previous generations of OCs, and are now widely used in most countries [18,19]. However, accumulating evidence suggests that new-generation OC preparations remain associated with the risk of CVD and related conditions, including stroke, myocardial infarction, and thromboembolism [20,21]. A meta-analysis of 18 studies showed that current modern OC use significantly increases the risk of ischemic stroke compared with non-use [22].

In a Danish cohort study of non-pregnant women aged 15–49 years, OCs containing high doses of ethinyl estradiol increased the risks of thrombotic stroke and myocardial infarction [23]. In a case–control study of 15–49-year-old women residing in the United Kingdom, current OC use was associated with an increased risk of venous thromboembolism (aOR = 2.97; 95% CI, 2.78–3.17) compared with OC non-use [24]. In another case–control study, the range of ORs for venous thromboembolism among OC users relative to non-users was 5.9–9.0, depending the duration of OC use [25].

Low-grade inflammation is an underlying cause of several chronic diseases, including CVD, and the role of hs-CRP as a biomarker for such inflammation has been reported widely [26,27]. In a Chinese cohort, elevated hs-CRP levels were significantly associated with the increased occurrence of major adverse cardiovascular and cerebrovascular events [28]. Elevated hs-CRP levels have been associated independently with increased CV risk in different clinical settings [29,30,31,32].

In this study, OC use significantly increased the frequency of risky (>1.0 mg/L) and high-risk (>3.0 mg/L) hs-CRP levels. Specifically, OC users were more likely than non-users to have hs-CRP levels >1.0 mg/L (aOR = 1.58; 95% CI, 1.25–1.98) and >3.0 mg/L (aOR = 1.51; 95% CI, 1.06–2.16); these two cutoff values are associated with the CVD risk. It was found that the use of OCs increased the risk of elevated hs-CRP levels, consistent with previous findings. In a study of young women (mean age, 23 years) in Italy, OC users had an OR of 4.04 (95% CI, 1.99–8.18) for high-risk (>3.0 mg/L) hs-CRP levels, which are associated with a high risk of CVD, compared with OC non-users [33]. In another study conducted in Italy with young sportswomen (mean age, 24 years), OC use significantly increased the OR for risky (OR = 10.9; 95% CI, 5.26–22.5) and high-risk (OR = 13.3; 95% CI, 4.14–42.6) hs-CRP levels compared with OC non-use [34]. The average age of our participants was 37.1 years; the difference between the OR values reported here and those obtained in the Italian studies may be due, at least in part, to the difference in the mean age of the study participants. In addition, racial/ethnic differences between Korean and Italian women, as well as differences in the hormonal constituents of the OCs used, likely influenced the OR magnitude.

To my knowledge, this study is the first to reveal the relationship between OC use and hs-CRP levels using nationwide survey data on premenopausal Korean women aged ≥19 years. However, this study has some limitations. Given the complexity of factors influencing serum hs-CRP levels, additional factors, including oxidative stress [35] and associated comorbidities, were likely not considered in the model established here. Factors such as the prevalence of CVDs or type 2 diabetes, use of anti-inflammatory drugs, and consumption of antioxidant foods or supplements need to be considered in future studies. Another study limitation is that the self-reporting of OC use and sociodemographic data, including alcohol consumption and smoking, may lead to misclassification or recall bias. Although the majority of OCs were third-generation, the types and amounts of hormonal components (estrogen and progestin) contained in OCs were heterogeneous. Therefore, this study has another limitation in that it does not take into account the type of OC or the actual length of time of taking OC. Despite these limitations, the study findings suggest that the possibility of chronic low-grade inflammation that can cause CVDs needs to be considered when selecting OC use as a family planning method.

## 5. Conclusions

In this study, conducted with nationally representative data on Korean women aged ≥19 years, OC use increased the low-grade inflammatory status, as reflected by hs-CRP levels. Increased chronic inflammation caused by OC use could increase the risks of cancer, diabetes, CVDs, and other inflammatory diseases in premenopausal women.

## Figures and Tables

**Table 1:** Demographic characteristics of the participants.

Characteristics	*n*	%
Age (Years)		
≤25	843	15.8
26–35	1314	24.6
36–45	2042	38.3
>45	1133	21.3
BMI		
<18.5	385	7.2
18.5–22.9	2816	52.8
23–25	905	17
>25	1226	23
Education		
<High School	261	4.9
High School	2083	39.1
>High School	2988	56
Average Household Income (USD/Month)		
<2500	1290	24.2
2500–3799	1357	25.4
3800–5600	1342	25.2
>5600	1343	25.2
Cigarette Smoking		
Yes	353	6.6
No	4979	93.4
Alcohol Drinking		
Yes	3314	62.2
No	2018	37.8

BMI: body mass index.

**Table 2:** Sociodemographic factors and hs-CRP levels by oral contraceptive (OC) use among Korean women.

Characteristics	OC User (*n* = 699)	Non-OC User (*n* = 4633)	*p*-Value ^1^
Age (Years), Mean (SD)	36.9 (9.2)	37.1 (9.3)	0.621
BMI, Mean (SD)	22.9 (3.9)	22.8 (3.7)	0.460
Average Household Income, USD (SD)	4072 (2562)	4254 (2438)	0.068
Education, *n* (% Within Group)			<0.001
<High School	67 (9.6)	194 (4.2)	
High School	278 (39.8)	1805 (39.0)	
>High School	354 (50.6)	2634 (56.9)	
Cigarette Smoking, *n* (% Within Group)			<0.001
Yes	83 (11.9)	270 (5.8)	
No	616 (88.1)	4363 (94.2)	
Alcohol Drinking, *n* (% Within Group)			0.642
Yes	440 (63.0)	2874 (62.0)	
No	259 (37.1)	1759 (38.0)	
hs-CRP, mg/L (SE)	1.087 (0.061)	0.953 (0.026)	0.045

^1^*p*-values for differences between the OC use and non-use groups were determined by the *t* test or Mantel–Haenszel chi-squared test. BMI: body mass index; SD: standard deviation; OC: oral contraceptive; hs- CRP: high-sensitivity C-reactive protein.

**Table 3:** Number of Korean women with risky and high-risk hs-CRP levels by oral contraceptive (OC) use.

Characteristics	OC User (*n* = 699)	Non-OC User (*n* = 4633)	*p*-Value ^1^
Risky hs-CRP, *n* (% Within Group)			<0.001
>1.0 mg/L	188 (26.9)	935 (20.2)	
≤1.0 mg/L	511 (73.1)	3698 (79.8)	
High Risk hs-CRP, *n* (% Within Group)			0.011
>3.0 mg/L	63 (9.0)	297 (6.4)	
≤3.0 mg/L	636 (91.0)	4336 (93.6)	

^1^*p*-values for differences between the OC use and non-use groups were determined by the Mantel–Haenszel chi-squared test. OC: oral contraceptive; hs- CRP: high-sensitivity C-reactive protein.

**Table 4:** Odds ratios and 95% confidence intervals for risky and high-risk hs-CRP levels by oral contraceptive (OC) use.

Model	Non-OC User	OC User	*p*-Value ^1^
Risky hs-CRP			
Crude	1.00 (Reference)	1.51 (1.23–1.86)	<0.001
Adjusted ^2^	1.00 (Reference)	1.58 (1.25–1.98)	<0.001
High-Risk hs-CRP			
Crude	1.00 (Reference)	1.65 (1.18–2.29)	0.003
Adjusted ^2^	1.00 (Reference)	1.51 (1.06–2.16)	0.024

^1^*p*-values were determined by multivariate logistic regression. ^2^ Adjusted for age, BMI, education, income, cigarette smoking, and alcohol consumption. OC: oral contraceptive; hs- CRP: high-sensitivity C-reactive protein.

## Data Availability

The data used to support the findings of this study are available from the corresponding author upon request.
